# Mental health challenges in health care workers during COVID pandemic

**DOI:** 10.1192/j.eurpsy.2023.1257

**Published:** 2023-07-19

**Authors:** S. Bilichodu Rangappa, S. Avula

**Affiliations:** 1 Psychiatry, Chamarajanagar Institute of Medical Sciences, Mysore, India; 2 Endocrinology, University of Minnesota, Eden Prarie, United States

## Abstract

**Introduction:**

Mental health issues of the health care workers (HCW) are often overlooked. It’s often presumed that situations like COVID are handled well by this group of population and hence their own mental wellbeing is ignored and neglected.

**Objectives:**

The objective of this study was to evaluate the depression and anxiety levels in healthcare workers who were on COVID duty.

**Methods:**

This study was performed over telephonic interview of all the healthcare workers who were performing COVID duty from March 2021 to December 2021. Hospital Anxiety and Depression Scale (HADS) was administered. Various factors influencing the presentation were then analyzed.

**Results:**

Over 534 healthcare workers were screened for psychiatric symptoms. About 76 fulfilled HADS criteria. 7.86% (42) scored above the anxiety cut off point and 6.36% (34) scored above the depression cut off point. About 373 (69.85%) expressed concerns about their safety and security as they were staying away from their families and about 469 (87.8%) expressed concerns about uncertainties about duty patterns.

**Conclusions:**

Health care workers should be screened for psychiatric illness if they are in constant stress. They should be well trained to carry out COVID duties which will reduce the anxiety about the duty patterns. Better awareness about COVID 19 may lead to decreased levels of anxiety and depression.

**Disclosure of Interest:**

None Declared

